# Image recombination transform algorithm for superresolution structured illumination microscopy

**DOI:** 10.1117/1.JBO.21.9.096009

**Published:** 2016-09-20

**Authors:** Xing Zhou, Ming Lei, Dan Dan, Baoli Yao, Yanlong Yang, Jia Qian, Guangde Chen, Piero R. Bianco

**Affiliations:** a Chinese Academy of Sciences, Xi’an Institute of Optics and Precision Mechanics, State Key Laboratory of Transient Optics and Photonics, No. 17 Xinxi Road, Xi’an, Shaanxi 710119, China; b Xi’an Jiaotong University, School of Science, No. 28 Xianning West Road, Xi’an, Shaanxi 710049, China; c University at Buffalo, Department of Microbiology and Immunology, No. 12 Capen Hall, Buffalo, New York 14214, United States

**Keywords:** fluorescence, structured illumination, superresolution

## Abstract

Structured illumination microscopy (SIM) is an attractive choice for fast superresolution imaging. The generation of structured illumination patterns made by interference of laser beams is broadly employed to obtain high modulation depth of patterns, while the polarizations of the laser beams must be elaborately controlled to guarantee the high contrast of interference intensity, which brings a more complex configuration for the polarization control. The emerging pattern projection strategy is much more compact, but the modulation depth of patterns is deteriorated by the optical transfer function of the optical system, especially in high spatial frequency near the diffraction limit. Therefore, the traditional superresolution reconstruction algorithm for interference-based SIM will suffer from many artifacts in the case of projection-based SIM that possesses a low modulation depth. Here, we propose an alternative reconstruction algorithm based on image recombination transform, which provides an alternative solution to address this problem even in a weak modulation depth. We demonstrated the effectiveness of this algorithm in the multicolor superresolution imaging of bovine pulmonary arterial endothelial cells in our developed projection-based SIM system, which applies a computer controlled digital micromirror device for fast fringe generation and multicolor light-emitting diodes for illumination. The merit of the system incorporated with the proposed algorithm allows for a low excitation intensity fluorescence imaging even less than $1\;W/{\mathrm{cm}}^{2}$, which is beneficial for the long-term, *in vivo* superresolved imaging of live cells and tissues.

## 1 Introduction

In the past decades, fluorescence microscopy has evolved toward superresolution imaging.^1^^–^^7^ Structured illumination microscopy (SIM),^8^^–^^11^ as one of the most promising superresolution techniques, possesses the merits of a high framing rate and low excitation intensity, compared to the stimulated emission depletion microscopy ($∼800\;\mathrm{MW}/{\mathrm{cm}}^{2}$),^12^ the reversible saturable optical fluorescence transition microscopy ($∼1.7\;\mathrm{MW}/{\mathrm{cm}}^{2}$),^13^ and the saturated SIM ($∼8\;\mathrm{MW}/{\mathrm{cm}}^{2}$).^14^ SIM in its linear form can increase the imaging resolution by a factor of two beyond the traditional wide-field microscopy when illuminating through the imaging objective lens. In this approach, a periodic sinusoidal fringe illumination with a spatial frequency close to the diffraction limit is adopted to shift the unresolved high-frequency features of the sample to the optical transfer function (OTF) domain of the objective lens. In addition, speckle-based SIM,^15^^,^^16^ instant SIM,^11^^,^^17^ and other forms of SIM^18^ are presently gaining increasing interest. These techniques further enrich the theory and address parts of the limitations of traditional SIM. Nevertheless, the maximum resolution extension of SIM is only twofold over the conventional microscopy. Further improvement of resolution can be realized by utilizing the nonlinear response of fluorescence molecules, such as saturated excitation (SE)^14^^,^^19^ or a photoswitchable (PS)^20^^,^^21^ mechanism. In practice, the SE-based nonlinear SIM requires high excitation intensity to satisfy the saturation condition that may harm the biological activity of living samples. For the PS-based nonlinear SIM, a relatively low intensity is permitted to activate the PS effect, but the photostability requirement for fluorescent dyes dramatically increases. Recently, the PS fluorescent protein was used in the PS-based nonlinear SIM technique developed by Betzig et al. to achieve 45- to 62-nm resolution.^21^ As the nonlinear SIM demands high excitation intensity or special fluorescent dyes with much more raw images required than the linear SIM, they also used a 1.7-NA objective to achieve 84-nm resolution under the linear total internal reflection fluorescence (TIRF)-SIM mode. Due to the advantages of low excitation power and high imaging speed, the linear SIM is still an ideal choice for dynamic imaging of living cells.

The generation and rapid phase control of high-quality fringe patterns is the key requirement in hardware for the SIM technique. This can be done by employing spatial light modulators (SLM), such as liquid crystal on silicon SLM, to generate adjustable orientation fringes by interference of two beams diffracted from the phase gratings addressed on the SLM.^22^ However, for maximal interference contrast, additional polarization control to maintain s-polarization for different fringe orientations is technically demanded, which often makes the optical configuration more complex. In addition, the high coherence of a laser beam inevitably produces speckle noises, which will seriously degrade the image quality. Although dynamic averaging of the speckle noise using a rotating diffuser or random waggling of the homogenizers such as a multimode fibers phase modulator will produce a smooth image, it will also limit the image acquisition speed and increase the complexity of the system. To address these limitations, we previously developed a micromirror device (DMD)-based light-emitting diode (LED)-illumination SIM system.^23^^,^^24^ In that approach, a binary fringe pattern loaded on DMD is demagnified and projected onto the specimen. The higher orders of spatial frequencies of the binary fringe are naturally blocked off due to the low-pass filtering effect of the objective lens, leading to a sinusoidal fringe illumination in the sample plane. The DMD projection and LED-illumination system have a few advantages, including no polarization control, high throughput, ease of multiwavelength switching, and free of speckle-noise. However, it should be noticed that the modulation depth of the sinusoidal fringe illumination projected on the specimen is determined by the OTF of the objective, which makes it decrease with the increase of the fringe frequency and the imaging depth. The low modulation depth of patterns will affect the quality of the final reconstructed images. So, the image reconstruction algorithm is an important issue for high-quality superresolution image retrieval. In this process, it is crucial to precisely determine the parameters of the fringe illumination pattern, especially the initial phase estimation.^25^ Incorrect initial phase estimation will seriously affect the superresolution result and leads to additional artifacts. Shroff et al.^26^ have presented a method for estimating the pattern phase by analyzing the phase of peaks (POP) of the delta function in the spectral space of spatial frequency of the captured image. It is a commonly used algorithm in superresolution image reconstruction for the linear SIM.^27^^,^^28^ However, for high-frequency or low modulation depth illumination patterns, this algorithm is less reliable. Wicker et al.^29^^,^^30^ have proposed two alternative methods based on iterative cross-correlation and noniterative auto-correlation reconstruction (ACR) algorithms, respectively. These algorithms predominantly circumvent the limitation of the POP method in the condition of a high-frequency illumination pattern. Nevertheless, for a low modulation depth illumination pattern, they did not provide a detailed discussion. In short, although the DMD-projection-based LED-illumination SIM system successfully addresses parts of limitation arising from the liquid crystal spatial light modulator-based laser-illumination SIM system, the optimal reconstruction algorithm still remains a question especially at low modulation depth of illumination patterns to keep the fidelity of the superresolution image.

In this paper, we propose a reconstruction algorithm based on an image recombination transform (IRT) scheme to determine the initial phase accurately and simplify the process of extracting the high-order spectral components as well. The precise solution of the initial phase can be obtained without any approximate conditions even at a very low modulation depth of fringe illumination. The IRT algorithm does not contain an iterative operation, which makes the whole reconstruction process fast and automatic. The validity of the IRT algorithm is demonstrated by imaging the bovine pulmonary arterial endothelial (BPAE) cells in our built DMD-projection-based, multicolor-LED-illumination SIM system. The merit of the system incorporated with the proposed algorithm allows for fluorescence imaging at excitation intensity as low as $1\;W/{\mathrm{cm}}^{2}$ using the LED light source, which represents a great decrease in excitation intensity compared to existing SIM systems (e.g., 30 to $100\;W/{\mathrm{cm}}^{2}$).^21^ The IRT algorithm can also be applied for the laser interference-based SIM as a substitution of the POP algorithm.

## 2 Theoretical Analysis

Considering a specimen characterized by a spatial distribution of fluorophores density S(r), it is illuminated by a cosine fringe intensity pattern I(r) with the form

$$I(r)=I_{0}[1+m\cdot \cos(2\pi p\cdot r+\varphi_{0})],$$ (1)

where p and $\varphi_{0}$ are the spatial frequency and the initial phase of the cosine fringe pattern, $I_{0}$ and m are the mean intensity and modulation depth, respectively. In linear SIM, the in-focus portion $S_{\text{in}}(r)$ of the specimen is modulated by the structured illumination patterns I(r) and the fluorescence emission intensity $E_{\text{in}}(r)$ is in linear response to the excitation light. Thus, we have

$$E_{\text{in}}(r)=I(r)\cdot S_{\text{in}}(r)=I_{0}[1+m\cdot \cos(2\pi p\cdot r+\varphi_{0})]\cdot S_{\text{in}}(r).$$ (2)

The captured fluorescence image by the detector can be described as a convolution of the emission fluorescence intensity and the point spread function (PSF) of the microscope. Thus, the in-focus image $D_{\text{in}}(r)$ formation is mathematically expressed as

$$D_{\text{in}}(r)=E_{\text{in}}(r)⊗H(r)=\{I_{0}[1+m\cdot \cos(2\pi p\cdot r+\varphi_{0})]\cdot S_{\text{in}}(r)\}⊗H(r),$$ (3)

where H(r) represents the PSF of the microscope and the symbol ⊗ denotes a convolution operation. Since the high spatial frequency excitation pattern attenuates very fast with defocus in the projection-type SIM, only the in-focus portion of the image is modulated, while the out-of-focus region is out of modulation.^31^ Therefore, the out-of-focus projection in the detected plane can be simplified as a nonmodulation item $B_{\text{out}}(r)$. Then, the final detected image can be rewritten as

$$D(r)=D_{\text{in}}(r)+B_{\text{out}}(r)=\{I_{0}[1+m\cdot \cos(2\pi p\cdot r+\varphi_{0})]\cdot S_{\text{in}}(r)\}⊗H(r)+B_{\text{out}}(r).$$ (4)

In the frequency domain, the spectrum of the detected image can be obtained by making a Fourier transform to Eq. (4)

$$\overset{˜}{D}(k)=I_{0}[{\overset{˜}{S}}_{\text{in}}(k)⊗\delta (k)+\frac{m}{2}{\overset{˜}{S}}_{\text{in}}(k)⊗\delta (k+p)e^{-i\varphi_{0}}+\frac{m}{2}{\overset{˜}{S}}_{\text{in}}(k)⊗\delta (k-p)e^{i\varphi_{0}}]\overset{˜}{H}(k)+{\overset{˜}{B}}_{\text{out}}(k)=I_{0}[{\overset{˜}{S}}_{w}(k)+\frac{m}{2}\cdot {\overset{˜}{S}}_{\text{in}}(k+p)e^{-i\varphi_{0}}+\frac{m}{2}\cdot {\overset{˜}{S}}_{\text{in}}(k-p)e^{i\varphi_{0}}]\cdot \overset{˜}{H}(k),$$ (5)

with ${\overset{˜}{S}}_{w}(k)=\frac{{\overset{˜}{S}}_{\text{in}}(k)\cdot \overset{˜}{H}(k)+{\overset{˜}{B}}_{\text{out}}(k)}{\overset{˜}{H}(k)}$.

The frequency distribution of ${\overset{˜}{S}}_{\text{in}}(k+p)$ and ${\tilde{S}}_{\text{in}}(k-p)$ is the unresolvable high-frequency features of the sample and shift to the support of the OTF, $\overset{˜}{H}(k)$. Generally, in the reconstruction procedure of SIM, three raw images are taken with the fringe phase shifted by 2π/3 between adjacent images. Combined with the deconvolution operation,^32^^,^^33^ the unresolved high-frequency features can be solved by Eq. (6) and only the frequency component ${\overset{˜}{S}}_{w}(k)$ contains the out-of-focus information

$$[\begin{array}{l} {\overset{˜}{D}}_{1}(k)\\ {\overset{˜}{D}}_{2}(k)\\ {\overset{˜}{D}}_{3}(k) \end{array}]=I_{0}H(k)[\begin{array}{ccc} 1 & \frac{m}{2}e^{-i\varphi_{0}} & \frac{m}{2}e^{i\varphi_{0}}\\ 1 & \frac{m}{2}e^{-i(\varphi_{0}+2\pi /3)} & \frac{m}{2}e^{i(\varphi_{0}+2\pi /3)}\\ 1 & \frac{m}{2}e^{-i(\varphi_{0}-2\pi /3)} & \frac{m}{2}e^{i(\varphi_{0}-2\pi /3)} \end{array}]\times [\begin{array}{l} {\overset{˜}{S}}_{w}(k)\\ {\overset{˜}{S}}_{\text{in}}(k+p)\\ {\overset{˜}{S}}_{\text{in}}(k-p) \end{array}].$$ (6)

To effectively extract the unresolved high-frequency information, the illumination pattern parameters of p, m, and $\phi_{0}$ should be precisely determined, especially for the initial phase $\phi_{0}$, because it is included in the exponential term of the matrix. A small estimation error of $\varphi_{0}$ will result in a serious artifact in the reconstructed image.

Shroff et al.^26^ have proposed the POP algorithm for estimating the initial phase, in which they analyze the value of the Fourier image at the pattern peak (i.e., k=p) with the form

$$\overset{˜}{D}(p)=I_{0}[{\overset{˜}{S}}_{w}(p)+\frac{m}{2}\cdot {\overset{˜}{S}}_{\text{in}}(2p)e^{-i\varphi_{0}}+\frac{m}{2}\cdot {\overset{˜}{S}}_{\text{in}}(0)e^{i\varphi_{0}}]\cdot \overset{˜}{H}(p).$$ (7)

It is noticed that when the out-of-focus background is weak, the frequency distribution of ${\tilde{S}}_{w}(k)$ is able to be approximated to the in-focus frequency distribution ${\tilde{S}}_{\text{in}}(k)$. If the power spectrum of the sample decreases sufficiently fast with growing frequency and also the modulation depth and the magnitude of OTF, $\tilde{H}(p)$, are sufficiently large, then the third term of Eq. (7) will be much larger than the remaining terms. In this case, we can ignore the small contribution of the first two terms in Eq. (7), and get the approximation of

$$\overset{˜}{D}(p)\approx \frac{m}{2}\cdot I_{0}{\overset{˜}{S}}_{\text{in}}(0)\overset{˜}{H}(p)\cdot e^{i\varphi_{0}}.$$ (8)

For any real valued samples and real PSF with symmetrical distribution, the center frequency component ${\tilde{S}}_{\text{in}}(0)$ and the OTF value $\tilde{H}(p)$ will be real. So, the phase contribution to Eq. (8) will only come from $e^{i\varphi_{0}}$. Hence, the initial phase can be estimated by solving the phase of this peak

$$\varphi_{0}\approx \arg[\overset{˜}{D}(p)].$$ (9)

The POP algorithm works well when all the above assumptions are fulfilled. For example, in the TIRF-SIM where the fluorescence excitation is generated in a thin volume with a depth typically below 200 nm, this excitation scheme has a very weak background and the POP algorithm can provide good precision in estimating the initial phase and separating the components well. However, in some cases, such as an existing strong background, or having a weak modulation depth or high-frequency illumination pattern, the POP algorithm cannot give an appropriate result.^29^^,^^30^

To address the limitation of the POP algorithm, here, we employ the phase shift between two adjacent images by π/2 rather than the commonly used value of 2π/3. Using Eq. (4), we can easily obtain the expression of the three captured images

$$D_{1}(r)=\{I_{0}[1+m\cdot \cos(2\pi p\cdot r+\varphi_{0})]\cdot S_{\text{in}}(r)\}⊗H(r)+B_{\text{out}}(r),D_{2}(r)=\{I_{0}[1-m\cdot \sin(2\pi p\cdot r+\varphi_{0})]\cdot S_{\text{in}}(r)\}⊗H(r)+B_{\text{out}}(r),D_{3}(r)=\{I_{0}[1-m\cdot \cos(2\pi p\cdot r+\varphi_{0})]\cdot S_{\text{in}}(r)\}⊗H(r)+B_{\text{out}}(r).$$ (10)

Because the intensity of the out-of-focus background $B_{\text{out}}(r)$ remains a constant, we can subtract two adjacent phase-shifted raw images to eliminate the background contribution

$$D_{12}(r)=D_{1}(r)-D_{2}(r)=\sqrt{2}m\cdot I_{0}\cdot \{[\cos(2\pi p\cdot r+\varphi_{0})\cos\frac{\pi }{4}+\sin(2\pi p\cdot r+\varphi_{0})\sin\frac{\pi }{4}]\cdot S_{\text{in}}(r)\}⊗H(r)=\sqrt{2}m\cdot I_{0}[\cos(2\pi p\cdot r+\varphi_{0}-\pi /4)\cdot S_{\text{in}}(r)]⊗H(r),$$ (11)

$$D_{23}(r)=D_{2}(r)-D_{3}(r)=-\sqrt{2}m\cdot I_{0}\cdot \{[\sin(2\pi p\cdot r+\varphi_{0})\cos\frac{\pi }{4}-\cos(2\pi p\cdot r+\varphi_{0})\sin\frac{\pi }{4}]\cdot S_{\text{in}}(r)\}⊗H(r)=-\sqrt{2}m\cdot I_{0}[\sin(2\pi p\cdot r+\varphi_{0}-\pi /4)\cdot S_{\text{in}}(r)]⊗H(r).$$ (12)

Then, we recombine a complex image by setting $D_{12}(r)$ as the real part and $D_{23}(r)$ as the imaginary part, respectively,

$$D_{c}(r)=D_{12}(r)-i\cdot D_{23}(r)=\sqrt{2}[m\cdot I_{0}e^{i\cdot (2\pi pr+\varphi_{0}-\pi /4)}\cdot S_{\text{in}}(r)]⊗H(r).$$ (13)

The Fourier transform of the recombined complex image is presented in the form

$${\overset{˜}{D}}_{c}(k)=\sqrt{2}me^{i\cdot (\varphi_{0}-\pi /4)}\cdot I_{0}{\overset{˜}{S}}_{\text{in}}(k-p)\cdot \overset{˜}{H}(k).$$ (14)

Substituting k=p into Eq. (14), we obtain

$${\overset{˜}{D}}_{c}(p)=A(p)\cdot e^{i(\varphi_{0}-\pi /4)},$$ (15)

where $A(p)=\sqrt{2}m\cdot I_{0}{\overset{˜}{S}}_{\text{in}}(0)\cdot \overset{˜}{H}(p)$. As discussed above, A(p) is real valued in the condition of real sample and symmetrical real PSF. Thus, the initial phase can be solved by

$$\varphi_{0}=\arg[{\overset{˜}{D}}_{c}(p)]+\pi /4.$$ (16)

It should be mentioned that if the noise is too high, the estimation will be disabled. After solving ${\tilde{D}}_{c}(p)$, one side of the unresolved high-frequency component can then be simply extracted by Eq. (14)

$${\overset{˜}{S}}_{\text{in}}(k-p)=\frac{e^{i\cdot \arg\{{\overset{˜}{D}}_{c}(p)\}}\cdot {\overset{˜}{D}}_{c}(k)}{\sqrt{2}mI_{0}\overset{˜}{H}(k)}.$$ (17)

Similarly, we can construct a conjugate image of the complex image $D_{c}(r)$. Then, the other side of the unresolved high-frequency component can also be extracted in the following expression:

$${\overset{˜}{S}}_{\text{in}}(k+p)=\frac{e^{i\cdot \arg[{\overset{˜}{D}}_{cc}(-p)]}\cdot {\overset{˜}{D}}_{cc}(k)}{\sqrt{2}mI_{0}\overset{˜}{H}(k)},$$ (18)

where ${\overset{˜}{D}}_{cc}(k)=\mathrm{FT}\{D_{c}^{*}(r)\}$, $\arg\{{\tilde{D}}_{c}(p)\}=-\arg\{{\tilde{D}}_{cc}(-p)\}-\pi /2$, FT and the symbol * denote the Fourier transform and the conjugation operation, respectively. Also, the wide-field component is easy to acquire by the Fourier transform of the sum of $D_{1}(r)$ and $D_{3}(r)$. In summary, the IRT algorithm first determines the initial phase by Eq. (16) and then subsequently retrieves the unresolved high-frequency components by Eqs. (17) and (18). After shifting the components to the correct positions and making deconvolution with the generalized Wiener filter, the final extended spectrum can be obtained, and then by taking an inverse Fourier transform, the superresolved image can be reconstructed.

Compared to the POP algorithm described by Eq. (9), the proposed IRT algorithm can provide a precise solution of the fringe initial phase without the assumptions of weak background and high modulation depth. In addition, the IRT algorithm can directly solve the unresolved in-focus high-frequency components of ${\overset{˜}{S}}_{\text{in}}(k+p)$ and ${\overset{˜}{S}}_{\text{in}}(k-p)$ by Eqs. (17) and (18), avoiding having to solve the matrix equation of Eq. (6).

## 3 Numerical Simulation

**Fig. 1:**
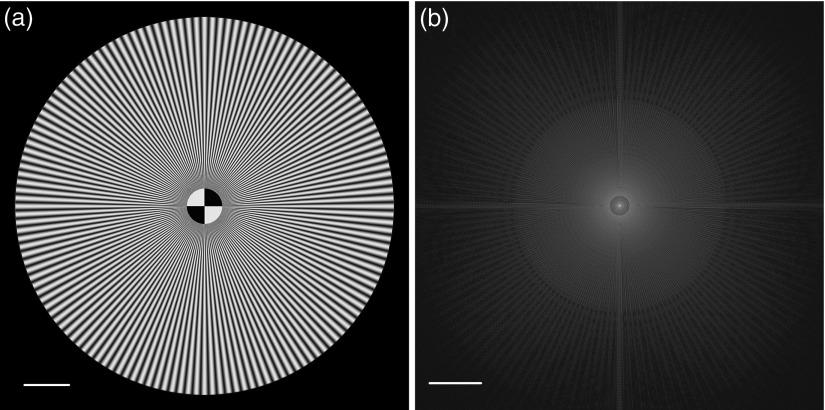
(a) Virtual object and (b) its Fourier transform spectrum. Scale bar: (a) 5  μm and (b) $2\;{\mu m}^{-1}$.

To verify the validity and feasibility of the IRT method, we first make a numerical simulation. In the simulation, the fringe illumination pattern is projected onto the sample through an oil-immersed objective (100×, NA=1.49, n=1.515). The fluorescence signal (emission wavelength at 461 nm) is collected by a digital camera (2048×2048  pixels, pixel size 6.5  μm×6.5  μm). The virtual object and its Fourier transform spectrum are shown in Figs. 1(a) and 1(b), respectively.

**Fig. 2:**
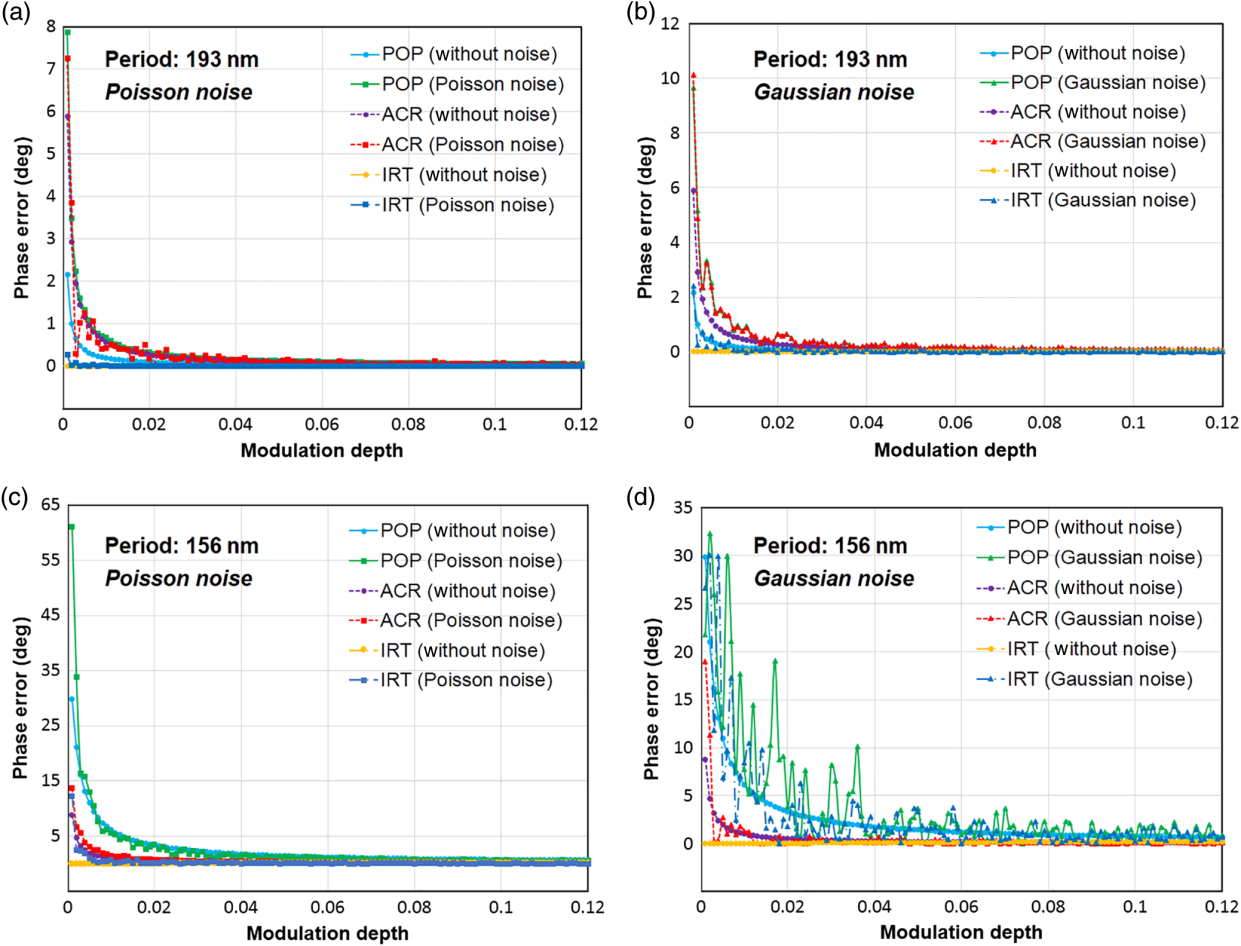
Simulation of initial phase estimation error under low modulation depth of illumination fringe at the period of (a) and (b) 193 nm and (c) and (d) 156 nm. For analyzing the influence of noises, the simulated images are degraded with (a) and (c) Poisson noise and (b) and (d) Gaussian noise with the variance of 0.001, respectively. In the presence of weak noises, the IRT algorithm exhibits excellent performance for low modulation depth.

According to the Abbe’s diffraction limit equation, the minimum period of the projection fringe is 155 nm at λ=461  nm with objective of NA=1.49. The initial phase estimation error under the condition of low modulation depth ranging from 0.001 to 0.12 with the fringe period of 193 and 156 nm are calculated by the POP, ACR, and IRT algorithms, respectively, as shown in Fig. 2. In theory, the phase error of the IRT algorithm should be zero in the absence of noise. The phase errors of the POP and ACR algorithms increase rapidly when the modulation depth is below 0.04 and 0.02, respectively, even without noise contribution, while the IRT algorithm always retains a relatively high precision over the whole range of the modulation depth. Considering Poisson noise is the predominant type of noise in low intensity images, we first add the Poisson noise in simulation [Figs. 2(a) and 2(c)] for comparing the performance of different algorithms. In this case, the maximal phase errors of the POP, ACR, and IRT algorithms read 7.9 deg, 7.3 deg, and 0.2 deg, respectively, at the period of 193 nm, corresponding to 80% of the maximum frequency supported by the OTF. When the fringe period decreases to 156 nm, approximating to the diffraction limit of the simulated system, the maximal phase errors of the POP algorithm increases up to 61 deg at the modulation depth of 0.001. On the other hand, the major contribution of electronic noise is mostly the Gaussian distribution. Thus, the comparison for the phase errors with Gaussian noise is also simulated in Fig. 2. The variances of the noise distribution are set to 0.001 [Figs. 2(b) and 2(d)]. Note that the sample spectrum signal is so weak at the OTF edge that the Gaussian noise becomes a major contribution. Therefore, the error curves fluctuate strenuously with the fringe period decreasing in the presence of Gaussian noise. In that condition, the IRT algorithm still can provide a relatively acceptable result, especially at the fringe period of 193 nm.

In short, for raw images with high modulation depth, all the algorithms are able to give a good performance in reducing the phase error. But in the condition of low modulation depth, only the IRT algorithm can estimate the initial phase with the best precision. However, similar to the other algorithms, if the noise is too strong, the IRT algorithm may not guarantee an acceptable precision of the initial phase.

**Fig. 3:**
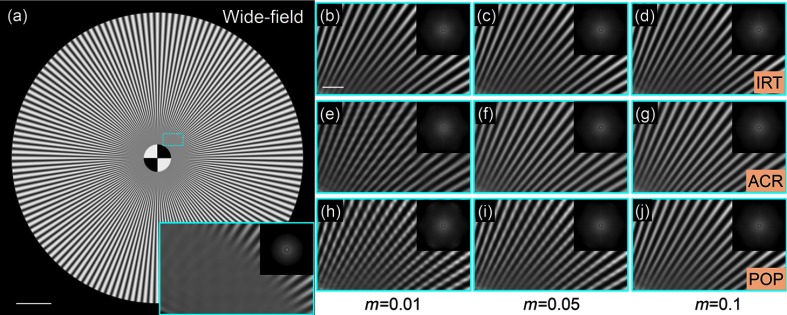
Reconstructed images of the simulated object using different methods at different modulation depth of fringe illumination. (a) The wide-field image and its magnified central part of the dashed boxed region. (b)–(g) The corresponding magnified superresolution images reconstructed by the IRT algorithm (b)–(d), the ACR algorithm (e)–(g) and the POP algorithm (h)–(j) at different modulation depth for comparison of the reconstructed image quality. The Fourier spectra of each reconstructed image are illustrated at the top-right corners, respectively. Compared to the wide-field image, all of the reconstruction methods are able to enhance the spatial resolution. However, with the decrease of the modulation depth, the reconstructed image by the POP algorithm produces more artifacts, while the IRT algorithm always retains a satisfactory performance. Scale bar: (a) 5  μm and (b)–(g) 500 nm.

To make a superresolution image reconstruction of SIM, three orientations of illumination fringes at 0 deg, 60 deg, and 120 deg are performed at the fringe period of 156 nm. The reconstructed images of the simulated object are shown in Fig. 3 with different algorithms at different modulation depth of fringe for comparison. Figure 3(a) is the wide-field image and its central region marked by a dashed box is magnified. Figures 3(b)–3(d) are the corresponding magnified superresolution images reconstructed by the IRT algorithm, Figs. 3(e)–3(g) and 3(h)–3(j) are corresponding to the ACR algorithm and the POP algorithm. The Fourier spectra of each reconstructed image are displayed at the top-right corners of the magnified images. It can be seen that all of the algorithms can successfully extend the support of the OTF in the frequency domain and enhance the spatial resolution of the image. However, with the decrease of the modulation depth, it is more and more difficult for the POP algorithm to separate the components well, and thus some residual errors come into being because of the estimation error of the initial phase. As previously speculated, owing to the incorrect spectrum expansion, a series of wave-like artifacts appear in the reconstructed images. This is particularly evident in Fig. 3(h). On the other hand, although the ACR algorithm is an effective method for phase estimation, the reconstruction process is time-consuming compared to the POP and IRT algorithms. Meanwhile, when the modulation depth is less than 0.01, a slight wave-like artifact also appears in the reconstructed image. In contrast, the IRT algorithm always retains a good performance even under low modulation depth, as seen in Fig. 3(b). When the modulation depth increases to more than 0.1, all of the algorithms give the same results.

## 4 Experiments and Results

**Fig. 4:**
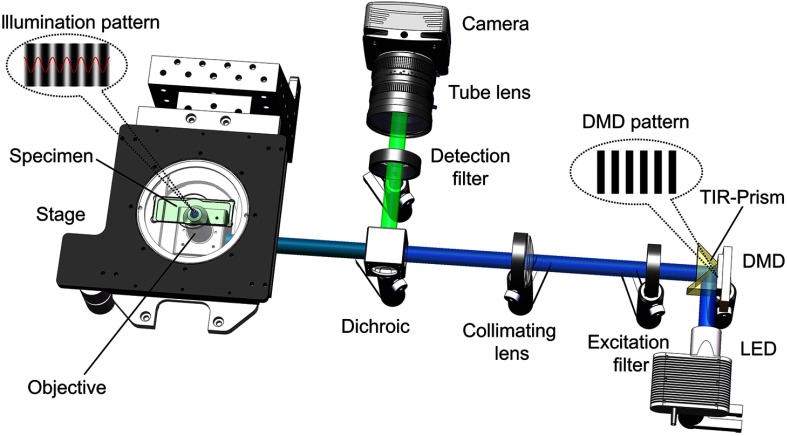
Schematic of the DMD-projection-based LED-illumination SIM system. LED: multiwavelengths of 405  nm/470  nm/565  nm/625  nm; DMD: maximal binary pattern rate of 32 kHz at 1024×768  pixels with a pixel pitch of 13.68  μm; excitation filter: quad-band bandpass of 390±22  nm/482±11  nm/563±7  nm/640±10  nm; dichroic: quad-edge reflection bands of 370 to 410  nm/473 to 491  nm/559 to 568  nm/633 to 647 nm; detection filter: quad-band bandpass of 446±18  nm/523±23  nm/600±20  nm/677±17  nm; objective: 100×/NA 1.49; camera: maximum frame rate of 100 fps at 2048×2048  pixels with a pixel pitch of 6.5  μm.

To further prove the applicability of the IRT algorithm, we used our developed DMD-projection-based LED-illumination SIM system^23^^,^^24^ to carry out the following experiment. The scheme of the SIM system is shown in Fig. 4. A four-wavelength high-power LEDs assembly (LED4D251, Thorlabs Inc.) with switchable wavelengths of 405, 470, 565, and 625 nm is employed as the illumination source for multiwavelength excitation. The LED light enters the TIR-Prism and is reflected to the DMD chip (DLP7000UV, Texas Instruments Inc.), where the fringe pattern is loaded. The light modulated by the DMD passes through a demagnifying optical system, consisting of a collimating lens, and a 100× objective (Apo TIRF, NA1.49, Nikon Inc., Japan), to produce a sinusoidal fringe illumination projected onto the specimen. A quad-band filter set (including a 390/482/563/640 nm quad-band bandpass excitation filter, a 446/523/600/677 nm quad-band bandpass emission filter, and a R405/488/561/635 nm quad-edge dichroic beamsplitter, Semrock Inc.) are used to separate the excitation lights and the emission fluorescence signals. A sCMOS camera with a maximum full-frame rate of 100 fps (Orca Flash4.0, 2048×2048  pixels, 16 bits gray depth, Hamamatsu Inc., Japan) is used to capture the two-dimensional image. The phase shifts of the fringe are controlled by the DMD chip without mechanical movement. If the fringe period is designed to occupy N pixels on the DMD chip, the minimum value of the phase shift will be 2π/N. Thus, to achieve a π/2 phase shift, just setting four pixels per period on the DMD chip, the illumination fringe phases corresponding to $\phi_{0}$, $\phi_{0}+\pi /2$, and $\phi_{0}+\pi$, will be obtained, respectively. In order to achieve a high imaging speed and a simplified pattern design, we apply only two perpendicular illumination fringe orientations in the experiment. Nevertheless, it is possible to generate multiorientation illuminations with specifically designed illumination patterns.

The photobleaching and phototoxicity effects have constantly been obstacles to long-term observation of living cells in fluorescence microscopy. The high excitation intensity will accelerate the photobleaching of fluorescent molecules and be harmful to specimens.^34^ The excitation intensities for the existing SIM systems (e.g., 30 to $100\;W/{\mathrm{cm}}^{2}$) are usually much stronger than that of the wide-field or light-sheet microscopies. In this experiment, we could image at intensities as low as $1\;W/{\mathrm{cm}}^{2}$, which is close to the life evolved condition ($0.1\;W/{\mathrm{cm}}^{2}$).^35^ This makes the system more suitable for bioimaging of low excitation intensities and long-term observation of specimens.

**Fig. 5:**
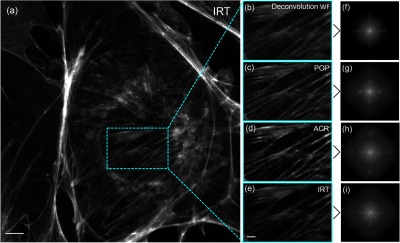
(a) The reconstructed superresolution image of F-actin in BPAE cells without artifacts by the IRT algorithm. For comparison, the magnified views of the dash-boxed region in (a) by different methods are, respectively, shown in (b)–(e), and the corresponding Fourier spectra are shown in (f)–(i). Some of residual components [indicated by pale-green circles in (g) and (f)] appear in the Fourier spectrum with the ACR algorithm and the POP algorithm, resulting in some unwanted information in the reconstructed image (c) and (d). In contrast, the IRT algorithm separates the components well in the Fourier spectrum (i), and thus effectively avoid artifacts and obtain a clear image (e). Scale bar: (a) 3  μm and (b)–(e) 1  μm.

**Fig. 6:**
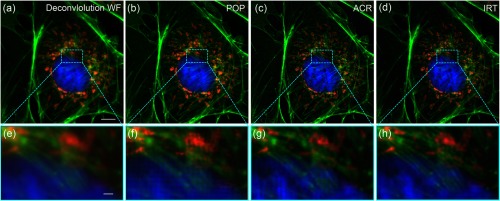
Superimposed images of the trichrome-stained BPAE cells reconstructed by different methods. (a) Deconvolution wide-field image, (b) superresolution image reconstructed by the POP algorithm, (c) superresolution image reconstructed by the ACR algorithm, (d) superresolution image reconstructed by the IRT algorithm. (e)–(h) The magnified views of the dash-boxed regions, respectively. Mitochondria are rendered in red, F-actins are rendered in green and nuclei are rendered in blue. Scale bar: (a)–(d) 5  μm and (e)–(h) 500 nm.

To demonstrate the feasibility of the IRT algorithm for the projection-based SIM, we employ the trichrome-stained BPAE cells (F36924, Thermo Fisher Scientific Inc.) as the specimen, which are labeled with MitoTracker Red CMXRos for Mitochondria, Alexa Fluor 488 phalloidin for F-actin, and DAPI for nuclei. The cells are sequentially illuminated using the excitation wavelengths of 405, 470, and 565 nm to achieve multicolor images. In this experiment, the modulation depth of the fringe is measured to be 0.046. Figure 5(a) shows a high-fidelity superresolution image of the F-actin obtained by the IRT algorithm. By applying the POP algorithm to process the same raw data, some unwanted residues appear in the Fourier spectrum as indicated by the pale-green circles in Fig. 5(g), resulting in some artifacts in the reconstructed image of Fig. 5(c). Compared to the POP algorithm, the ACR algorithm obtains an improvement in artifacts suppression and the residual fringes are hardly seen in its reconstructed image [Fig. 5(d)]. But there still exist a few extra components in the Fourier spectrum [Fig. 5(h)], leading to the reduction of image quality. In terms of computation time, the POP, ACR, and IRT algorithms take 2.97, 9.65, and 3.02 s, respectively, to calculate one superresolution image from six raw images (2048×2048  pixels) using a personal computer (Intel Core i7-4790K 4 GHz processor and 32 GB RAM) with the MATLAB software (R2013a) operating under Windows 7 (SP1) x64. Similar experimental results of superimposed tricolor images of BPAE cells are shown in Fig. 6. Here, the mitochondria are rendered in red, the F-actins are rendered in green, and the nuclei are rendered in blue. By the POP algorithm, the F-actin and mitochondria exhibit some extra stripes in the reconstructed images [Figs. 6(b) and 6(f)]. These artifacts may confuse the scientific evaluation of biological morphology. However, in the IRT superresolution images [Figs. 6(d) and 6(h)], such residual stripes are not observed and the image qualities are better than the ACR images shown in Figs. 6(c) and 6(g). This once again proves that the IRT algorithm is a robust and high-accuracy reconstruction method for superresolution imaging.

Although the IRT algorithm is immune to the modulation depth of the illumination fringe, it should be noticed that the noise in the captured structured illumination images is another challenge to the accurate determination of the initial phase. In fact, when the image noise is too high, Eq. (15) cannot be strictly tenable, because the noise contribution must be considered. As a result, the solution of the initial phase of Eq. (16) will contain a phase error. So, obtaining a high signal-to-noise ratio (SNR) of structured illumination images is crucial for increasing the accuracy of initial phase determination. In our system, we use a high sensitivity sCMOS camera with 16 bits gray depth to capture high SNR images, which guarantees obtaining high-fidelity superresolution images. Based on the potential advantages of the DMD-projection-based LED-illumination SIM system, incorporated with the proposed IRT superresolution algorithm, future work will be extended to fast multicolor live-cell imaging and object tracking.

## 5 Conclusion

We have proposed an IRT superresolution image reconstruction algorithm for SIM. Compared to the traditional POP algorithm and the ACR algorithm, the IRT algorithm can obtain the initial phase of fringe with high precision, and extract the high-order spectral components without matrix operation. Numerical simulations show that the IRT algorithm always keeps a higher precision of initial phase estimation than the POP algorithm. The immunity of the IRT algorithm against the modulation depth of fringe patterns and the high precision of initial phase determination enable reconstructing an artifact-free superresolution image as long as the SNR of the captured images is reasonably high. We have proved the feasibility and reliability of the IRT algorithm by employing it in our built DMD-projection-based, multicolor-LED-illumination SIM system to image the BPAE cells at low excitation intensity. The experimental results demonstrate that the IRT method can effectively avoid artifacts to produce high-fidelity superresolution images. The IRT algorithm is applicable in either the projection-based SIM or the interference-based SIM.
